# Community dynamics and co-occurrence relationships of pelagic ciliates and their potential prey at a coastal and an offshore station in the ultra-oligotrophic Eastern Mediterranean Sea

**DOI:** 10.3389/fgene.2023.1219085

**Published:** 2023-07-20

**Authors:** Filomena Romano, Paraskevi Pitta, Uwe John

**Affiliations:** ^1^ Marine Biological Section, University of Copenhagen, Helsingør, Denmark; ^2^ Hellenic Centre for Marine Research, Institute of Oceanography, Heraklion, Greece; ^3^ Ecological Chemistry, Alfred Wegener Institute for Polar and Marine Research, Bremerhaven, Germany; ^4^ Helmholtz Institute for Functional Marine Biodiversity at the University of Oldenburg (HIFMB), Oldenburg, Germany

**Keywords:** barcoding, DNA, mixotrophy, network analysis, prey-predator, vertical/temporal distribution

## Abstract

Ciliates have been recognized as one of the major components of the microbial food web, especially in ultra-oligotrophic waters, such as the Eastern Mediterranean Sea, where nutrients are scarce and the microbial community is dominated by pico- and nano-sized organisms. For this reason, ciliates play an important role in these ecosystems since they are the main planktonic grazers. Regardless the importance of these organisms, little is known about the community structure of heterotrophic and mixotrophic ciliates and how they are associated to their potential prey. In this study, we used 18S V4 rRNA gene metabarcoding to analyze ciliate community dynamics and how the relationship with potential prey changes according to different seasons and depths. Samples were collected seasonally at two stations of the Eastern Mediterranean Sea (HCB: coastal, M3A: offshore) from the surface and deep chlorophyll maximum (DCM) layers. The ciliate community structure varied across depths in HCB and across seasons in M3A, and the network analysis showed that in both stations, mixotrophic oligotrichs were positively associated with diatoms and showed few negative associations with ASVs annotated as marine Stramenopiles (MAST). On the other hand, heterotrophic tintinnids showed negative relationships in both HCB and M3A stations, mostly with Ochrophyta and Chlorophyta. These results showed, in first place that, although the two stations are close to each other, the ciliate dynamics differed between them. Moreover, mixotrophic and heterotrophic ciliates may have different ecological niches since mixotrophic ciliates may be more selective compared to heterotrophic species regarding their prey. These findings are the first glimpse into an understanding of the dynamics between heterotrophic and mixotrophic ciliates and their role in microbial assemblages and dynamics of ultra-oligotrophic environments.

## 1 Introduction

Microbial planktonic eukaryotes are major components of the marine food web, covering different trophic modes from heterotrophy to mixotrophy and autotrophy, and therefore contributing to the biogeochemical cycling of the oceans (Pomeroy, 1974; Azam et al., 1983; Flynn et al., 2019). Studying the dynamics and diversity of the microbial community contributes remarkably to our understanding of the ecological process and ecosystem function, including the microbial food web, especially in ultra-oligotrophic waters where nutrients are scarce (Poindexter, 1981; Labiosa et al., 2003; Siokou-Frangou et al., 2010). Inside the microbial food web components, ciliates have been recognized as one of the major group of the microbial loop (Azam and Malfatti, 2007; Fenchel, 2008; De Vargas et al., 2015; Bai et al., 2020; Wang et al., 2020; Liu et al., 2021) since they are major grazers of nano-picoplankton and also because they are a major food source for larger zooplankton, including copepods and fish larvae (Song et al., 2009; Hu et al., 2019; Huang et al., 2021).

These roles are particularly critical in ultra-oligotrophic environments like the Mediterranean Sea, which is characterized by a gradient of increasing oligotrophy from west to east (Turley, 1999; Barale et al., 2008; D’Ortenzio and D’Alcalà, 2009). The east basin demonstrates qualities such as high transparency, low nutrient levels, and low productivity (Azov, 1986; Yacobi et al., 1995; D’Ortenzio and D’Alcalà, 2009).

In this environment, the microbial community is mostly dominated by pico-sized organisms rather than large photo-autotrophs like diatoms (Berman et al., 1984; Christaki et al., 2001; Pitta et al., 2001; Tanaka et al., 2007). For this reason, ciliates are considered to be the main planktonic grazers in the Mediterranean Sea (Pitta and Giannakourou, 2000; Pitta et al., 2001). Previous studies based on morphological analysis (Prog et al., 1989; Pitta and Giannakourou, 2000; Bojanić et al., 2001; Pitta et al., 2001; Rekik et al., 2015; Romano et al., 2021) (Jonsson, 1987; Prog et al., 1989; Suzuki and Taniguchi, 1998; Pitta and Giannakourou, 2000; Bojanić et al., 2001; Rekik et al., 2015; Romano et al., 2021) or on molecular approaches like high-throughput sequencing (Pawlowski et al., 2012; Stoeck et al., 2014; Santoferrara et al., 2017) focusing on ciliate dynamics show that, within the class Spirotrichea, the subclasses Oligotrichia and Choreotrichia (Lynn, 2008) dominate the marine planktonic ciliates in the oligotrophic waters.

In the last decades, many studies about the composition and structure of the microbial food web, especially of ciliates, were carried out in both marine and freshwater environments since they provide powerful tools for measuring the impacts of pollution and other human activities on the coastal environments. Thus, a high taxonomic level of ciliate species identification is needed.

For a long time, planktonic ciliate diversity and species taxonomy were mostly explored morphologically through microscopy using classical phytoplankton fixatives, like Lugol or formaldehyde, in case mixotrophy needs to be taken into account (Suzuki and Taniguchi, 1998; Romano and Pitta, 2021; Romano et al., 2021), and, more recently, through more demanding methods, like Protargol impregnation (Wilbert, 1975 Foissner, 2014; Ji and Wang, 2018). However, morphospecies identification is very time-consuming and difficult as it requires substantial experience in sample preparation and taxonomical training. Moreover, the microscopy approach does not allow for the recognition and precise species identification of very small organisms or of those with few or similar morphological characteristics, or it cannot distinguish cryptic diversity that remains, most of the time, unexplored.

However, the diversity of the ciliate community has been extensively investigated in recent times due to the technological development of molecular tools that have expanded our capacity to better understand the structure of the microbial food web (Caron, 2016; Leray and Knowlton, 2016). These new molecular approaches have been applied in many different ecosystems, from islands to oceanic and coastal waters and extreme environments (De Vargas et al., 2015; Ainsworth et al., 2017). New studies conducted in many different environments showed that the microbial community as a whole, and more specifically the ciliate community, is extremely diverse in both coastal and oceanic waters (De Vargas et al., 2015; Massana et al., 2015).

In the Mediterranean Sea, most of the studies on ciliate metabarcoding analysis focused mainly on the geographical differentiations of the benthic community (Santoferrara et al., 2014; Ganser et al., 2021) and the characterization of parasitic ciliate species (Scheifler et al., 2019).

Using high-throughput sequencing and DNA metabarcoding, together with ecological information, our study aimed to: 1) assess the diversity of the ciliate community in two different stations of the ultra-oligotrophic Eastern Mediterranean Sea, one coastal (HCB) and one off-shore (M3A); 2) investigate the seasonal and vertical dynamics of the ciliate community at the two stations; and 3) identify their potential prey and their relationship with the other taxa using co-occurrence matrix and network analysis.

## 2 Methods

### 2.1 Study area and sampling strategy

The Cretan Sea is part of the Eastern Mediterranean basin and one of the most oligotrophic environments (Siokou-Frangou et al., 2010; Christaki et al., 2011). Two stations, a coastal (HCB: 35°43′42″N; 25°07′92″E) and an offshore (M3A: 35°72′63″N; 25°13′07″E) one, were sampled in March, May, July and October 2019; M3A station was not sampled in May due to rough sea. Both stations are part of the Poseidon system, a monitoring system for the Greek Seas. In both stations, water was sampled at two different depths: surface (2 m) and DCM (according to the season, usually between 50 and 100 m). Temperature and chlorophyll a (Chla) were measured using a Seabird CTD profiler at seven different depths in the euphotic layer (2, 10, 20, 50, 75,100 and 120 m). Nutrients (DIN = dissolved inorganic nitrogen and PO_4_^3-^) were measured according to Strickland and Parsons (1972) and Rimmelin and Moutin (2005), respectively.

### 2.2 Filtration, DNA extraction and sequencing

Equal volumes of water samples (10 L) were collected and pre-filtered on a 150 μm mesh-size net to remove most of the macro-zooplankton, and then the water was filtered onto cellulose polyester 0.2 μm pore size filters (142 mm, ®Millipore) using a peristaltic pump and they were cut in four pieces and immediately frozen in liquid nitrogen and later stored at −80°C until DNA extraction. Total DNA from each filter was extracted using the DNeasy 96 PowerWater Kit (^®^ Qiagen) according to the manufacturer’s instructions. DNA concentration was measured by Nanodrop (®Thermofisher) and the DNA samples were stored at −20°C until further analysis. The hypervariable V4 region of the eukaryote SSU rDNA gene was amplified using the following primers: V4F_Illumina and V4R_illumDiv + Hapto (5′ CCAGCASCYGCGGTAATTCC-3′ and 5′ ACT​TTC​GTT​CTT​GAT-3′). The performed PCR reactions had a total volume of 25 μL, containing 2.5 µL of microbial DNA (5 ng μL^-1^), 5 µL of both amplicon forward and reverse primers (1 µM) and 12.5 µL of high-fidelity polymerase (®Kapa Biosystems). Plates were sealed and the following PCR-program was run in a thermal cycler: initial denaturation at 95°C for 3 min, followed by 25 cycles of 95°C for 30 s, annealing at 55°C for 30 s; extension at 72°C for 30 s, and a final extension step at 72°C for 5 min. The following library preparation of 18S rRNA gene amplicons was performed: PCR clean-up 1, index PCR, PCR clean-up 2, library quantification, normalization and pooling following the 18S Metagenomic Sequencing Library Preparation guide^1^. Library denaturing and sample loading to the Illumina MiSeq system were carried out to perform a 2 × 300 bp paired-end sequencing. Raw sequences were deposited in the Sequence Read Archive (https://www.ncbi.nlm.nih.gov/sra) under the BioProject PRJNA990967 (https://www.ncbi.nlm.nih.gov/bioproject/PRJNA990967).

### 2.3 Bioinformatic pipeline

The raw reads were cleaned and merged with DADA2, v1.16. (Callahan et al., 2017) and reads were subsequently classified with “assignTaxonomy”, the DADA2 implementation of the naive Bayesian classifier method.

For taxonomic annotation, we used the Protist Ribosomial Reference Database PR2 v 4.12.0 (Guillou et al., 2013).

We removed from the dataset all taxa that did not belong to eukaryotic unicellular plankton (including fungi, cnidarians, and metazoans), and excluded singletons and doubletons.

The clean dataset resulted in ∼6 millions reads that were grouped in 5,042 total amplicon sequence variants (ASVs). Trophic strategies were manually annotated and curated based on the currently accepted forms of protistan plankton nourishment (Flynn et al., 2019; Schneider et al., 2021).

Based on their trophic strategy, protists were divided into autotrophs, mixotrophs, heterotrophs and unknown (NA), according to Schneider et al. (2021).

### 2.4 Statistical analyses

All statistical analyses and plots were conducted in R 4.0.2 (R Core Team, 2018). For multivariate and comparative analyses, the abundance dataset was rarefied with a random sub-sampling to the lowest number of sequences (n = 321) with the “rrarefy” function, R package vegan version 2.5–5 (Oksanen et al., 2011), and ASVs with less than 100 reads were removed to avoid bias in the statistical analysis. The ciliate dataset was then filtered by the total one for specific analysis. Alphadiversity of the ciliate community was determined using Shannon H′ and Pielou’s Evenness indices as indicators for ciliate biodiversity, and the number of Observed ASVs as an estimation of species richness (Shannon, 1948; Pielou, 1966).

Further analyses were performed using the “phyloseq” package (McMurdie and Holmes, 2013).

Samples were classified as winter (March), spring (May), summer (July) and autumn (October), while depths were divided in two categories: surface and DCM. Nonmetric multidimensional scaling (NMDS) was performed using the “metaMDS” function (Oksanen et al., 2011) based on a Bray–Curtis dissimilarity matrix (Bray and Curtis, 1957) using an ASV table modified following the Hellinger transformation.

Furthermore, we examined the distribution of samples using the principal component analysis (CCA) on our ciliate diversity data. Ciliate community *β* diversity between seasons, months and depths was tested with MANOVA on the Bray-Curtis distance of Hellinger-transformed ASV tables. To assess protists’ ecological niches at different depths, ASVs were classified as “depth generalists” and “depth specialists” using the “clamtest” function in the vegan package version 2.5–5 (Oksanen et al., 2011). The clamtest was conducted on both HCB and M3A datasets. Based on the abundance in the two stations and a specialization threshold K), the clam multinomial model classified taxa into one of four groups: 1) depth generalist; 2) surface specialist; 3) DCM specialist; and 4) too rare to classify with confidence. As K, we applied a conservative threshold, as in Minicante et al. (2019).

Co-occurrence matrices were calculated with the “cooccur” package in R (Griffith et al., 2016) for both stations, to understand the negative and positive associations between ciliates and their potential prey. Correction for multiple-testing of the *p* values was performed in R according to the Benjamini–Hochberg method (Benjamini and Hochberg, 1995). Correlations were then sorted for statistical significance using the “prob.table” function in the cooccur package. The relation coefficients from the co-occurrence matrix, together with the ecological niche specialization for every ciliate and potential prey species, were used to build a network analysis.

Gephi software version 0.9.7 (Bastian et al., 2009) was used to calculate the network topological parameters, such as the clustering coefficient as the measure of the degree to which nodes in a graph tend to cluster together and the graph density as the measure for the proportion of possible relationships in the network.

## 3 Results

### 3.1 Environmental variables

**FIGURE 1:**
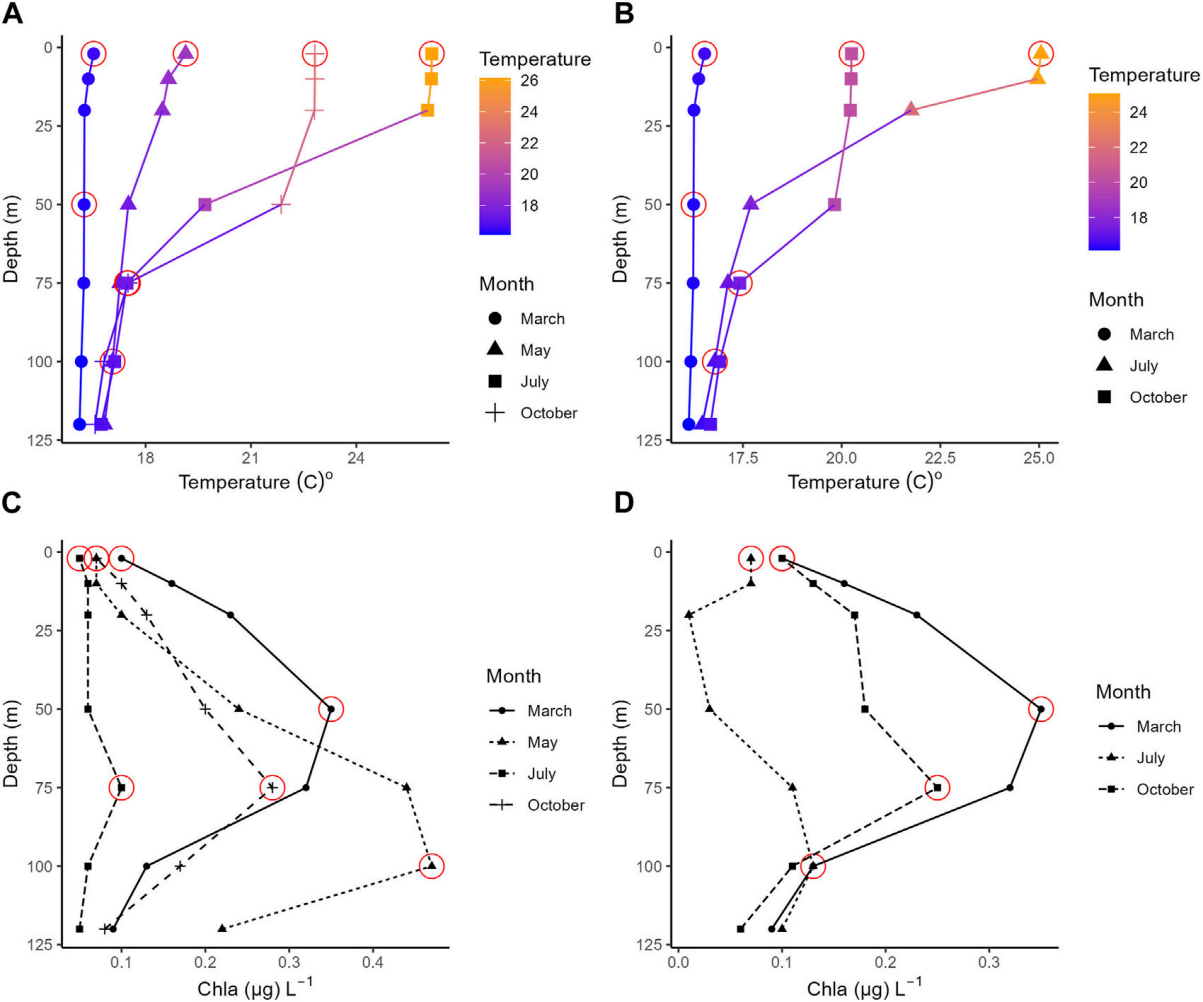
Vertical profile of temperature and chlorophyll a in HCB **(A,C)** and M3A **(B,D)**. Depths with red circles are the one sampled for metabarcoding analysis.

The two sampling sites are shown in Supplementary Figure S1. The environmental factors varied across seasons and depths (Supplementary Table S1). Water temperature had the same range in both stations; it reached the minimum value in winter at the surface (15.4°C in HCB and 15.3°C in M3A), while the highest temperatures in HCB and M3A were found in summer at surface (26.2°C in HCB and 25.0 in M3A, Figures 1A, B). Chl *a* concentration showed the same range in both HCB and M3A. It was higher in winter and lower in summer at the surface at both stations (0.51–0.05 μg L^-1^ in HCB; 0.43–0.05 μg L^-1^ in M3A, Figures 1C, D).

DIN concentration ranged from 1.92 to 0.32 μM in HCB, with the highest value at the surface during winter and the lowest one at the surface during summer. On the other hand, DIN concentration in M3A ranged from 1.42 to 0.55 μM, with the highest value at DCM during summer and the lowest at the surface during autumn.

Inorganic PO_4_^3-^ varied across the two stations. In HCB, the highest value was detected at the surface in winter (90.33 nM), while the lowest one was detected at the surface in autumn (2.11 nM). In M3A, the highest phosphate value was detected at the surface in summer (26.58 nM) and the lowest one at the surface in autumn (2.10 nM, Supplementary Table S1). Differences between the seasons were only significant for Chla (*p* < 0.05), while differences between depths were only significant for temperature (*p* < 0.05). The two stations did not show any significant differences for any of the environmental variable.

### 3.2 Seasonal variability of the microbial food web components

Our dataset consisted of 14 samples (8 for HCB and 6 for M3A) and 14,228,752 raw V4 18S rRNA gene sequence reads for HCB and 2,360,611 for M3A. The filtering procedure generated a final dataset, including 16,589,363 total sequence reads that, after normalization, numbered 3,129,215. After DADA2 processing, there were 5,031 ASVs at 97% similarity, of which 485 were annotated to Ciliophora.

The assigned trophic modes, read abundances and taxonomic assignments of all the groups are in Supplementary File S1. Rarefaction curves indicated saturated sampling for all samples (Supplementary Figure S2).

**FIGURE 2:**
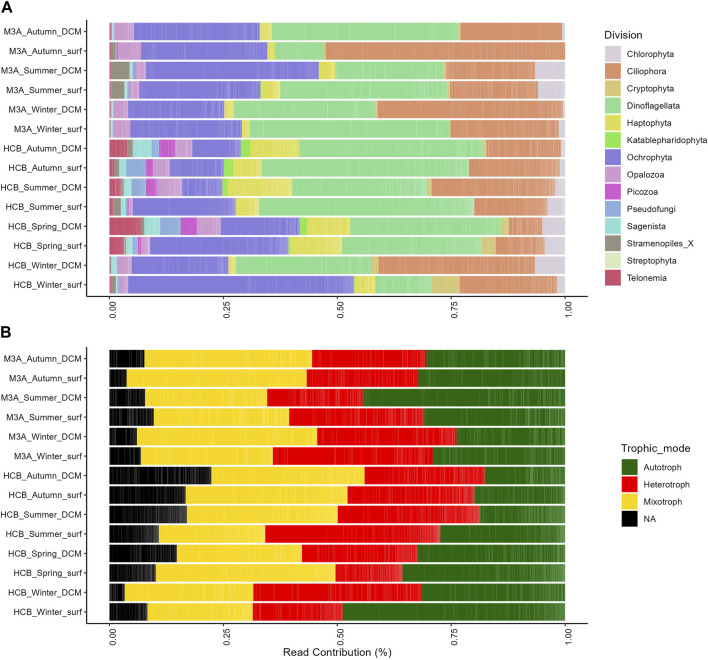
Relative abundance of reads for all the components of the microbial food web **(A)** and percentage of ASVs of heterotrophic, mixotrophic and autotrophic protists **(B)** in all samples.

The seasonal and vertical composition of eukaryotic groups varied across samples. Dinoflagellata, Ochrophyta and Ciliophora were the most abundant groups in the entire dataset in terms of number of reads (43.67%, 18.96% and 17.58%, respectively), followed by Haptophyta (5.87%), Chlorophyta and Opalozoa (∼2%) and by Telonemia, Pseudofungi, Cryptophyta, Sagenista, Stramenopiles, Picozoa, Katablepharidophyta, Streptophyta and Centroheliozoa (<2%). At the coastal station HCB, Dinoflagellata dominated the autumn, spring and summer samples, while Ochrophyta were more abundant during winter in terms of percentage of reads; on the other hand, at the offshore station M3A, Dinoflagellata dominated all samples with the only exception of the surface layer during autumn, where the highest percentage of Ciliophora was recorded (49.22% of total number of reads). Other groups showed relatively low contribution in terms of reads to all samples. For example, the group Haptophyta was more abundant in HCB compared to M3A and the highest relative abundance was recorded during spring at the surface and at the DCM in summer (Figure 2A). For the trophic mode distribution across the samples, most of the autotrophic protists were recorded during the winter and summer, at the surface in HCB and at DCM in M3A, respectively. On the other hand, heterotrophic protists made ∼30% in all samples, with the exception of summer at DCM in M3A, and of winter at the surface in HCB. Mixotrophic protists, instead, contributed for ∼40% in all samples with the exception of the surface in spring and at DCM in winter regarding HCB, while in M3A the highest contribution was found during autumn at the surface. Most of ASVs that could not be annotated as autotrophic, mixotrophic or heterotrophic protists (NA) were mostly found in HCB spring, summer and autumn, with the highest contribution at the DCM in autumn (Figure 2B).

### 3.3 Ciliate distribution

A complete species list of all ciliates is reported in Table 1. The total number of ciliate ASVs varied across samples and they accounted for an average of 15.47% of the total number of ASVs per sample. The highest percentage of ciliate ASVs was detected in winter at the surface of M3A (offshore station, 19.92%) and the lowest was detected in autumn at the DCM of M3A (offshore station, 4.89%, Table 2).

**TABLE 1:** List of the number of ASVs detected for all the ciliates species, together with the total number of reads and the presence (+) or absence (///) in HCB and M3A.

Species	ASVs	Reads	HCB	M3A
*Leegaardiella_*sp.	73	63546	+	+
*Strombidiidae_H_X_*sp.	35	48702	+	+
*Unknown*	77	39185	+	+
*Pseudotontonia_*sp.	22	26682	+	+
*Pelagostrobilidium_*sp.	19	16698	+	+
*Strobilidiidae_I_X_*sp.	11	16698	+	+
*Strombidiida_D_XX_*sp.	9	15630	+	+
*Tintinnida_XX_*sp.	13	14798	+	+
*Lynnella_semiglobulosa*	4	10197	+	+
*Tontoniidae_A_X_*sp.	4	10029	+	+
*Strombidiidae_L_X_*sp.	13	8597	+	+
*Strombidiida_F_XX_*sp.	3	8168	+	+
*Strombidiidae_J_X_*sp.	19	7924	+	+
*Stenosemella_pacifica*	2	7025	+	+
*Eutintinnus_*sp.	2	6769	+	///
*Tontoniidae_B_X_*sp.	6	6072	+	+
*Eutintinnidae_X_*sp.	5	6016	+	+
*Strombidiidae_G_X_*sp.	6	6008	+	+
*NASSO_1_*sp.	25	5850	+	+
*Tintinnidae_X_*sp.	5	5324	+	+
*Pseudotontonia_simplicidens*	6	5040	+	+
*Askenasia_*sp.	11	4934	+	+
*Strombidiida_G_XX_*sp.	1	4835	+	+
*Strombidiidae_Q_X_*sp.	3	4008	+	+
*Didiniidae_X_*sp.	2	3887	+	+
*Strombidiidae_B_X_*sp.	4	3263	+	+
*Salpingella_*sp.	6	3232	+	+
*Undellidae_X_*sp.	5	3049	+	+
*Dadayiella_ganymedes*	1	3046	+	+
*Parastrombidinopsis_*sp.	1	2826	+	+
*Strombidiidae_M_X_*sp.	2	2620	+	+
*Amphorides_quadrilineata*	4	2614	+	+
*TIN_03_X_*sp.	1	2606	+	+
*Steenstrupiella_steenstrupii*	5	2586	+	+
*Rhabdonella_spiralis*	1	2349	+	+
*Strombidiida_XX_*sp.	7	2310	+	+
*Parallelostrombidium_conicum*	1	1948	+	+
*Codonellopsis_*sp.	2	1933	+	+
*Eutintinnus_medius*	2	1692	+	+
*Leegaardiellidae_A_X_*sp.	5	1640	+	+
*Strombidium_*sp.	3	1538	+	///
*Epiplocylis_undella*	2	1454	+	+
*Spirotontonia_*sp.	2	1450	+	+
*Tintinnidium_*sp.	3	1356	+	+
*Strombidium_caudispina*	1	1325	+	+
*Strombidium_M_*sp.	2	1279	+	+
*Rimostrombidium_A_*sp.	2	1118	+	+
*Dictyocystidae_X_*sp.	1	1038	+	+
*Eutintinnus_apertus*	2	987	+	///
*Peritromus_kahli*	2	775	+	+
*Strombidium_capitatum*	3	773	+	+
*Amphorellopsis_*sp.	2	754	+	+
*Dictyocysta_*sp.	2	552	+	+
*Varistrombidium_kielum*	1	528	+	///
*Eutintinnus_fraknoi*	3	497	+	+
*Strombidium_triquetrum*	2	471	+	///
*Tiarina_*sp.	2	469	+	+
*Codonaria_cistellula*	1	458	///	+
*Xystonella_longicauda*	1	365	+	+
*Strombidinopsis_*sp.	2	351	+	+
*Urotricha_*sp.	2	346	+	///
*Strombidinopsis_batos*	1	330	+	///
*Strombidiidae_O_X_*sp.	1	314	+	+
*Strombidinopsis_acuminata*	1	309	+	///
*Amphorellopsis_acuta*	1	305	+	///
*Salpingacantha_*sp.	2	291	+	+
*Ptychocylis_*sp.	1	280	+	///
*Salpingella_acuminata*	4	244	+	///
*Discotrichidae_X_*sp.	2	239	+	///
*Strombidiida_B_XX_*sp.	1	197	+	+
*Pelagostrobilidium_neptuni*	1	184	+	+

**TABLE 2:** Total number of ASVs detected in this study, together with the total number and the percentage of ciliate ASVs in each sample. The total average of the percentage of ciliate ASVs is reported in bold.

Sample	N^o^ total ASVs	N^o^ ciliate ASVs	% Ciliate ASVs
HCB_Winter_surf	1162	165	14.20
HCB_Winter_DCM	820	159	19.39
HCB_Spring_surf	1718	206	11.99
HCB_Spring_DCM	1130	125	11.06
HCB_Summer_surf	1210	174	14.38
HCB_Summer_DCM	1963	209	10.65
HCB_Autumn_surf	1333	236	17.70
HCB_Autumn_DCM	1414	123	8.70
M3A_Winter_surf	507	101	19.92
M3A_Winter_DCM	748	145	19.39
M3A_Summer_surf	1003	157	15.65
M3A_Summer_DCM	717	118	16.46
M3A_Autumn_surf	367	118	32.15
M3A_Autumn_DCM	634	31	4.89
		**Average**	**15.47%**

Among Ciliophora, the orders Choreotrichida, Strombidiida and Tintinnida dominated the ciliate dataset and they were the most abundant ones in terms of percentage of reads detected (Supplementary Table S2). The highest percentages of the order Strombidiida was detected at the offshore station M3A (55.47%), during spring (61.20%) and at the surface layers (48.59%), followed by Choreotrichida, which were more abundant at the coastal station HCB (31.89%), during summer (35.05%) and at the DCM (42.21%). Tintinnida, instead, were also more abundant at the coastal station HCB (21.08%) but more abundant during winter (32%) and at the surface (23.73%) Supplementary Table S2). The group “Other ciliates”, which comprises representatives of the classes Nassophorea and Prostomatea from the CONThreeP supercluster as well as from the class Heterotrichea, were detected in a lower abundance in terms of reads and numbers of ASVs compared to the class Spirotrichea. The total number of ASVs and reads were 56 and 20,853, respectively. All the orders were more abundant in HCB compared to M3A in terms of reads. Choreotrichida dominated in summer, with *Leegaardiella* sp. as the most abundant species at the DCM and *Lynnella semiglobulosa* and Strobilidiidae_I_X_sp. at the surface. At the offshore station M3A, Choreotrichida were more abundant in autumn, with the species *Leegaardiella* sp. and *Pelagostrobilidium* sp. dominating the surface. Also Strombidiida were more abundant at the coastal station HCB, where they were mostly recorded in summer and autumn, with *Pseudotontonia* sp. and Strombidiidae_H_X_sp. that dominated both the surface and the DCM. These two species were also mostly abundant in autumn at the surface at the offshore station M3A. Strombidiida was the other with the most of unknown ASVs that were recorded in summer at the surface of HCB.

**FIGURE 3:**
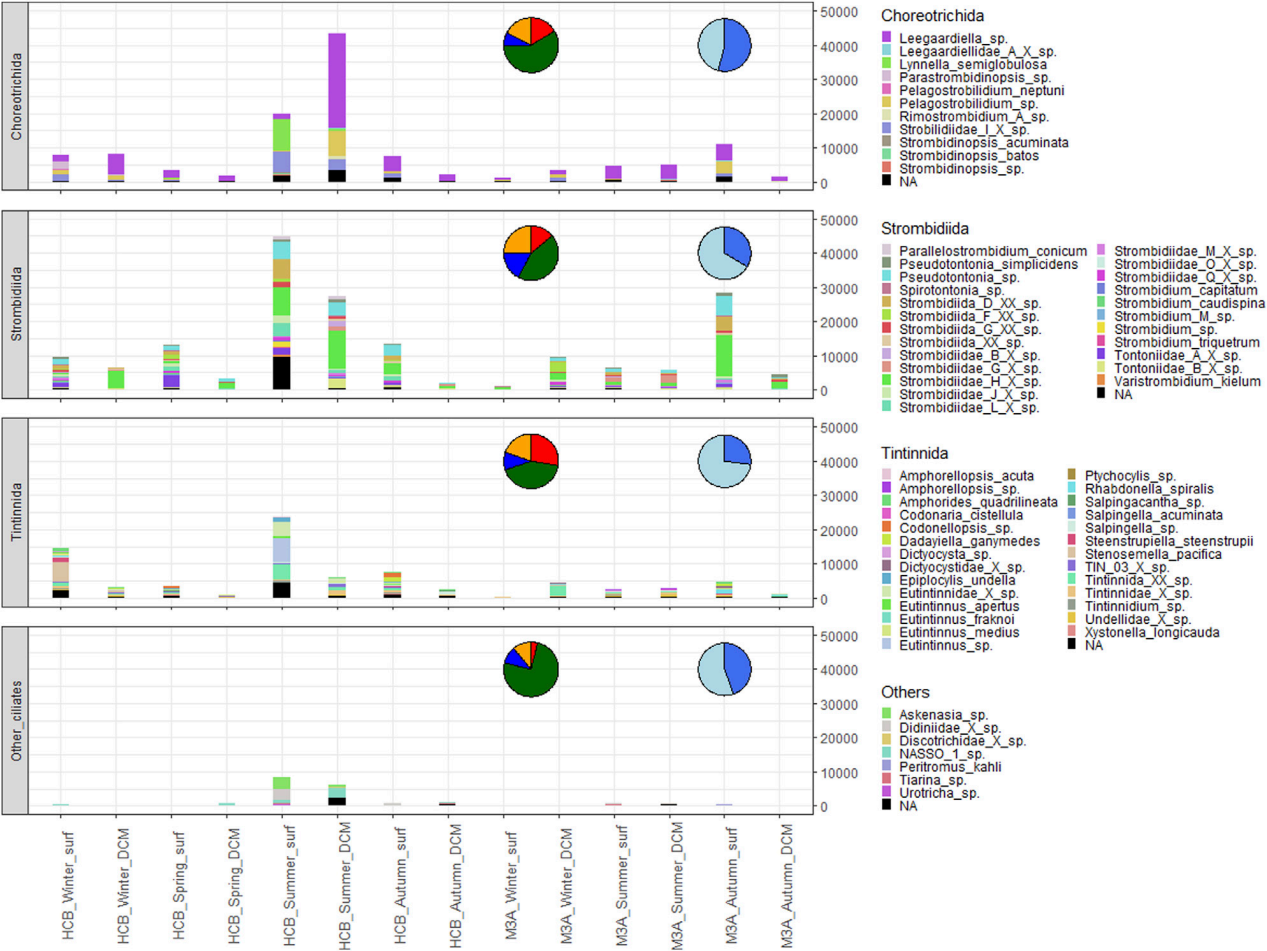
Total number of reads of the orders Choreotrichida, Strombidiida, Tintinnida (Spirotrichea) and “Others” in all the samples. Pie charts represent the percentage of reads recorded in summer (green), autumn (yellow), winter (red) and spring (dark blue) on the left, and between surface (light blue) and DCM (blue) on the right one.

On the other hand, Tintinnida were detected in HCB, in summer and in winter. The surface sample in winter was mostly dominated by the species *Streenstrupiella streenstrupii* and *Stenosemella pacifica*. The surface sample in summer instead, showed a different Tintinnida composition, with species of *Eutintinnus* genus being the most dominant ones (Figure 3). The orders Choreotrichida and Tintinnida, which together they represent more than 50% of the total reads, are characterized by only heterotrophic species. For this reason, most samples were dominated by heterotrophic ciliates rather than mixotrophic ones, which can be found only within the order Strombidiida. The highest percentage of heterotrophic ciliates was found in winter at the surface at both HCB (70.63%) and M3A (60.20%), while the lowest percentage was found in spring at HCB (34.96%) and in autumn at M3A (36.72%) at the surfaces, where consequently, the highest percentages of mixotrophic ciliates were detected (HCB Spring surface = 64.10%; M3A Autumn surface = 63.28%, Table 3), followed by the summer season, where the percentage of mixotrophic ciliates was ∼40% of the total ciliate dataset at both HCB and M3A.

**TABLE 3:** Relative contribution of heterotrophic, mixotrophic and unknown trophic ciliates in all samples.

Sample	Heterotrophs	Mixotrophy	Unknown
HCB Winter surface	70.63	29.21	0.16
HCB Winter DCM	63.49	36.51	0
HCB Spring surface	34.96	64.10	0.94
HCB Spring DCM	52.39	46.45	1.16
HCB Summer surface	53.69	44.13	2.18
HCB Summer DCM	66.80	32.98	0.22
HCB Autumn surface	54.06	44.04	1.89
HCB Autumn DCM	70.52	29.04	0.44
M3A Winter surface	60.20	39.80	0
M3A Winter DCM	45.09	54.91	0
M3A Summer surface	54.18	45.25	0.57
M3A Summer DCM	59.11	40.89	0
M3A Autumn surface	36.72	63.28	0
M3A Autumn DCM	45.09	54.91	0

The highest relative abundance of unknown trophic modes was found in summer at the surface of HCB (2.18%).

### 3.4 Alpha and beta diversity of the samples based on the ciliate community

The observed number of ASVs was higher in HCB compared to M3A (MANOVA *p* < 0.01**, Supplementary Table S3). The highest values, 272 and 167, were found in autumn and in summer at the surface in HCB and M3A, respectively. The lowest number was observed in spring (142) and autumn (31) at DCM in HCB and M3A, respectively. On the other hand, Shannon and Pielou’s Evenness indices fell in the same range in both HCB and M3A (4.53–3.98 in HCB; 4.37–2.72 in M3A, and 0.84–0.75 in HCB; 0.87–0.76 in M3A, respectively). The highest Shannon values were found also in autumn and in summer at the surface in HCB and M3A, respectively, while the lowest were found during summer at surface in HCB and during autumn at DCM in M3A. In the HCB station, the evenness was also lower in summer at the surface and higher in autumn at the DCM, while in M3A the lowest evenness value was found during winter at the DCM, and it increased until the pick in autumn at the surface (Supplementary Table S4).

Generally, the observed number of ASVs and Shannon indexes, as indicators for ASV abundance and ASV richness, respectively, were higher at the surface in all seasons in HCB, with the exception of summer (Supplementary Figure S3). On the opposite side, the observed ASVs at the M3A station were higher at DCM compared to the surface layer except in summer. Shannon and Evenness indices, in contrast, were always higher at the surface layer in M3A.

NMDS showed a clear separation of the two stations in summer and especially in autumn. Only in winter were the stations rather similar to each other (Supplementary Figure S4).

**FIGURE 4:**
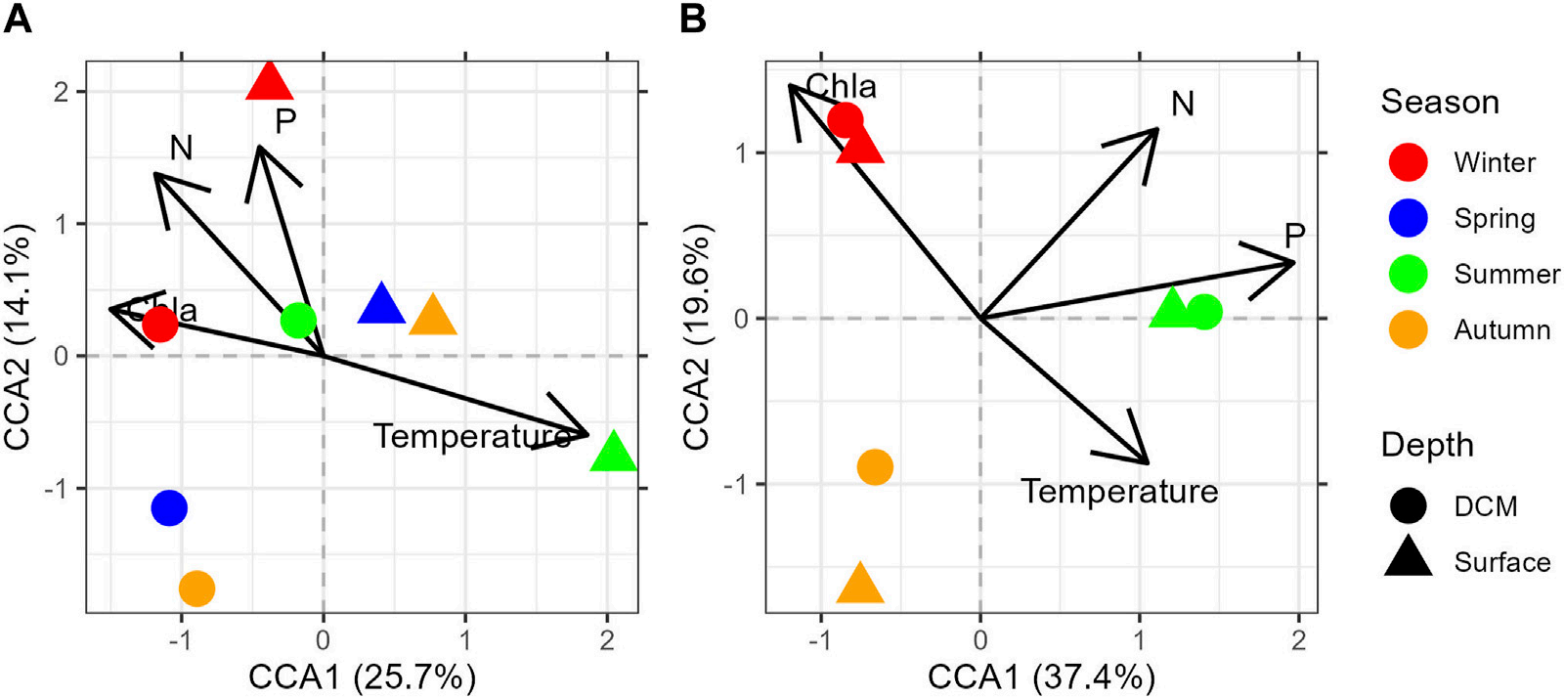
CCA conducted in HCB **(A)** and M3A **(B)** on total ciliate community.

CCA identified temperature and oxygen as statistically significant variables (*p* < 0.001***), followed by Chl *a* (*p* < 0.05*). In HCB, the separation of samples was based on depth and in M3A on seasons (Figures 4A, B).

For HCB, the first two canonical axes explain 38.5% of the total variance (Figure 4A), while for M3A, the first two canonical axes explained 57% of the total variance (Figure 4B).

The HCB samples separated along the temperature gradient that was negatively correlated with the other variables (Chl *a,* DIN, PO_4_^3-^ and Oxygen, Figure 4A). For this reason, the samples are separated into surface samples, more related to the temperature gradient, and DCM samples that are more related to the distribution of nutrients and Chl *a*. M3A samples separated clearly according to the seasons. Summer samples in M3A were correlated to the amount of PO_4_^3-^ while winter samples were correlated to Chla (Figures 4A, B).

### 3.5 Ecological niches: depth generalists vs depth specialists

The CLAM test conducted for the HCB surface and DCM samples generated a complete dataset of the whole microbial food web, and counted 32% of the total ASVs classified as surface specialists, the 38% classified as DCM specialists, 28% as generalists and only 2% classified as too rare for analysis. In total, 70% of total ASVs were classified as specialists for one of the two depths rather than generalists. In M3A, on the other hand, most of the ASVs (38%) were classified as too rare, followed by 36% classified as generalists. Only the 17% and 9% of the total ASVs were classified as surface and DCM specialists, respectively (Supplementary File S1, Supplementary Figure S5). ASVs classified as surface specialists in HCB belonged to the groups Ochrophyta and Chlorophya, while the DCM specialists were annotated as Haptophyta and Opalozoa. In M3A instead, these groups were annotated as generalists.

For ciliates, most mixotrophic ASVs were annotated as surface specialists, followed by 49 DCM specialists, 34 generalists and 5 rare, in HCB. On the other hand, most of the heterotrophic ciliate ASVs were classified as DCM specialists (102), followed by 99 surface specialists, 45 generalists and 3 rare.

In M3A, most of the ASVs were classified as generalists or too rare for analysis, such as mixotrophic ASVs (69) and 126 heterotrophic ASVs, respectively.

### 3.6 Co-occurrence between ciliates and potential prey

The co-occurrence analysis conducted on the HCB dataset produced a network with 138 nodes and 300 edges, of which 218 were positive and 72 were negative. The statistics showed that the clustering coefficient was 0.981, and the path length 1.029. Moreover, the graph density was 0.032.

On the other hand, the network analysis conducted on the M3A station produced only 42 nodes and 308 edges, of which 240 were positive and 68 were negative. The clustering coefficient was higher compared to HCB; while the average path length was lower (1 for both).

In HCB, most of the ciliates with the highest degree numbers were depth generalists. Most of the connections were found between Ciliophora (20.29% of the total nodes) and Ochrophyta (50.17% of the total nodes). All mixotrophic ciliates showed positive connections with diatoms and other species belonging to the Ochrophyta division, except for Strombidiida_B_XX_sp., which showed negative relationships with *Chaetoceros elegans* and *Chaetoceros muellerii*. Other mixotrophic ciliates, like *Strombidium caudispina*, were positively connected to *Pelagococcus* sp., *Chrysophyceae* sp. and the diatom *Chaetoceros rostratus*. *Parallelostrombidium conicum*, on the other hand, showed a positive relationship with Rhizochromulinales and Sarcinochrydiaceae. Heterotrophic ciliates, such as *Amphorellopsis acuta, Bergeriella ovata* and *Parastrombidinopsis* sp., showed negative connections with diatoms. Chlorophyta (9.42% of the total nodes) showed few connections with ciliates. Also in this case, mixotrophic ciliates, like *P. conicum* and Strombidiida_B_XX_sp., showed positive relationships with the species of these divisions, while some tintinnids (*Eutintinnus* sp., *Dadayiella ganymedes* and *S. pacifica*) had negative relationships with *Micromonas* sp. and *Ostreococcus* sp.

**FIGURE 5:**
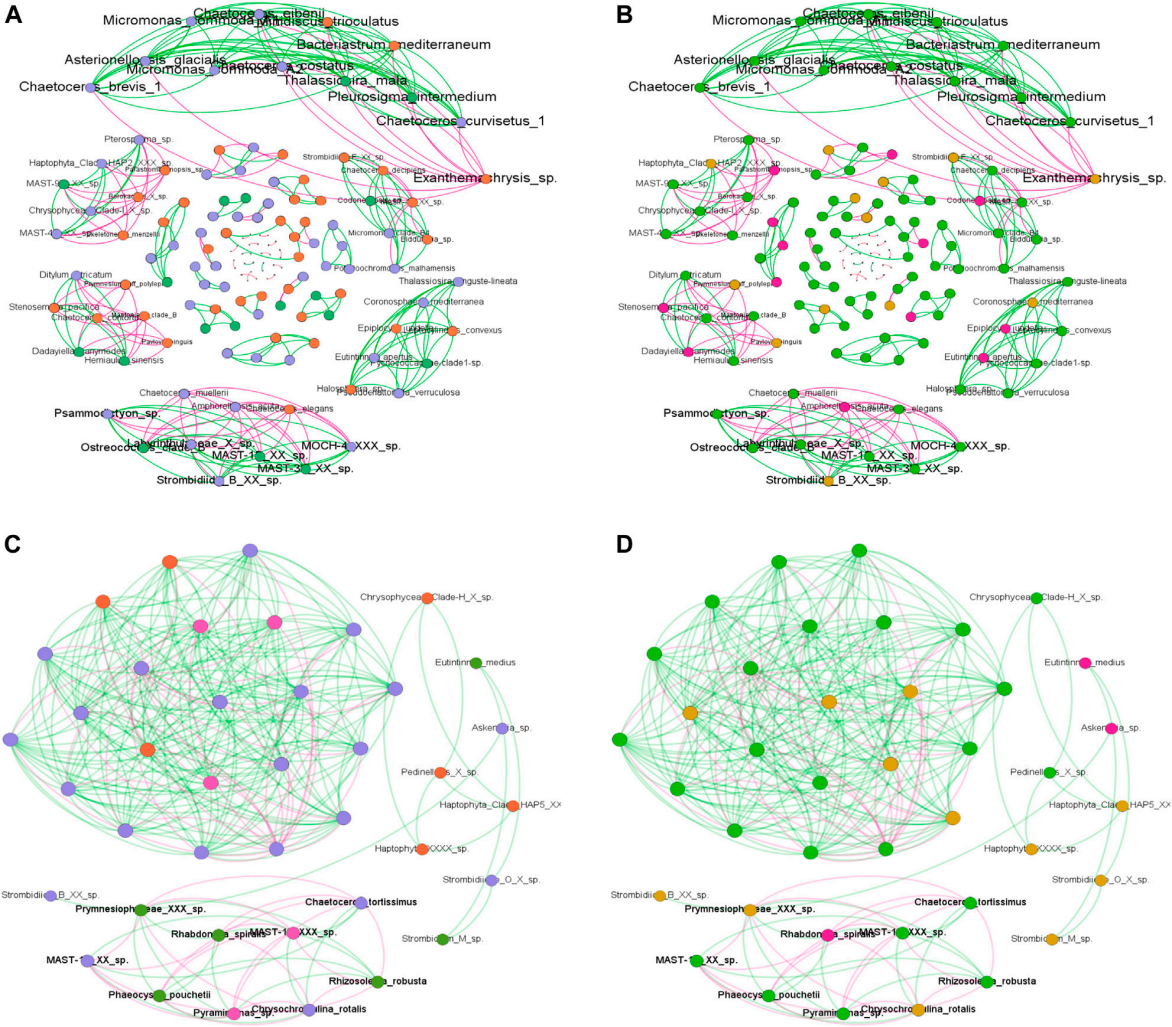
Network analysis conducted in HCB **(A–C)** and M3A **(B–D)** on the total dataset. **(A–C)** represent the network analysis conducted in HCB and M3A respectively using the results of the clam test as node annotations (Purple = depth generalists; green = surface specialists; orange = DCM specialists). **(B,C)** are the networks conducted in HCB and M3A, respectively using the trophic stage as node annotations (Green = autotrophs; yellow = mixotrophs; pink = Heterotrophs). Green and red edges represent the positive and negative connections in all the networks.

Mixotrophic ciliates showed negative correlations only with Stramenopiles and Streptophyta (Figures 5A–D).

The M3A network was more complex compared to the HCB one. Six ciliate ASVs were considered for this analysis, of which three of them were annotated as surface specialists (*Eutintinnus medius*, Strombidium_M and *Rhabdonella spiralis*) and the other three were annotated as depth generalists (Strombidiidae_B, Strombidiidae_O and *Askenasia* sp.). The most complex part of the network did not include any ciliate ASVs; instead the connections showed positive and negative relationships between diatoms and other components of the picoplankton. The heterotrophic ciliates *Askenasia* sp. and *Rhabdonella spiralis* showed positive connections with Prymnesiophyceae_XXX_sp., while mixotrophic ciliate species did not show any connection with any of the potential prey components (Figures 5A–D).

## 4 Discussion

### 4.1 V4 region of 18S rRNA gene in microplanktonic community

In the present study we used universal primers by Stoeck et al. (2010) with slight modification of (Piredda et al., 2017) to compensate for the bias against Haptophytes. These primers were designed and identified by manual inspection of an alignment of over 1000 eukaryotes SSU rDNA sequences including environmental samples, and are used in many of metabarcoding studies targeting marine protist including ciliates.

It is true that some groups in the present study, such as dinoflagellates, made the highest contribution to the community in most of the samples and that has probably most to do with its large genomes and high copy numbers of the rRNA genes, For this reason, absolute read abundance comparisons with metabarcoding data are often problematic, in particular between different groups. However, we are convinced that a general bias of our primers in favour of dinoflagellates and in disfavor of ciliates is unlikely, as some samples (Autumn surface in M3A, and Winter surface in HCB) were not dinoflagellate dominated using the same primers. Moreover, unknown taxa belonging to different microplanktonic groups showed different dynamics through the sampling period. Therefore, our results were most likely a reflection of the unique ecology at our sampling site and not caused by a bias in the molecular method used. Moreover, rarefaction analysis confirms the saturated sampling profiles for all samples so the observed difference in ciliate community is not due to undersampling.

### 4.2 Two stations close enough but different

Using 18S V4 rRNA metabarcoding coupled with Illumina sequencing, we assessed the seasonal and vertical dynamics of pelagic ciliates in relation to additional components of the microbial food web. This study represents the first dataset from the ultra-oligotrophic Mediterranean Sea focused on the seasonality of pelagic ciliates using metabarcoding, coupled with a network analysis to have a preliminary overview of prey-predator co-occurrences in two different ecological niches represented by surface and DCM.

The samplings were carried out in two different stations: a coastal one, which is supposedly more affected by the anthropogenic activities, and an offshore station. Despite the geographical distance between the two stations being small, we could assess differences in the ciliate community, as the dynamics were affected according to the depth at the coastal station, and according to season in the offshore one. Since coastal stations are easily accessible, and the maximum depth is recorded at around 200 m, a good body of literature exists on aloricate ciliates in coastal environments based on morphospecies (Cariou et al., 1999; Agatha, 2011; Ganser et al., 2021), while metabarcoding data are mostly available from benthic ciliate species or only on tintinnids (Santoferrara et al., 2014; Massana et al., 2015; Tucker et al., 2017). Little is known about ciliate dynamics in offshore environments, especially in the Mediterranean Sea, where the maximum depth can reach down to 1500 m, like in this study.

Our study shows, although only at two sample stations, that the ciliate community compositions and dynamics are different in HCB and M3A; ciliates are more susceptible to the depth differences in HCB compared to M3A, which showed a higher seasonal heterogeneity in the community composition. For example, *Leegardiella* genus was higher always at the DCM compared to the surface in HCB, whereas in M3A, the distribution was different according to seasons (higher in summer in both surface and DCM compared to winter and autumn). Another example is the Strombidiidae_H_X genus, which was higher at DCM compared to surface in all seasons in HCB, but this genus reached the highest abundance during autumn in both surface and DCM in M3A.

These differences in ciliate dynamics in two different, although close, stations were found also in New England waters where, despite a comparable mixing in the surface layers at both stations, the community composition showed contrasting patterns (Tucker et al., 2017).

These results were also supported by the *β* diversity of the samples in the CCA, where the samples from HCB were separated by depth, with all surface samples correlated with the temperature gradient, and the DCM samples correlated with nutrients. On the other hand, the M3A samples were separated by season whereas surface and DCM correlated to the same environmental variables.

### 4.3 Difference in ciliate dynamics and correlations with abiotic factors

The analysis of the V4 hypervariable region of the 18S rRNA gene in the two stations of the Cretan Sea conducted in four seasons and at two depths provides the first detailed temporal overview of planktonic protists in an area where morphology-based studies on these communities date back to the 19th century.

During the last decades, studies on pelagic ciliates were conducted using classical inverted microscopy. Forty-seven microzooplankton taxa (Bandelj et al., 2008) were identified in light microscopy, which is possibly a gross underestimation of planktonic ciliate diversity given that we detected more than 400 ciliate-assigned ASVs in just two stations. Considering that our dataset only included two stations, the actual diversity of planktonic ciliates is likely even greater than the one observed in this study.

The results obtained from this study are comparable with other metabarcoding studies conducted in the Mediterranean Sea, but there is a lack of literature on the ultra-oligotrophic Eastern part (Minicante et al., 2019; Santi et al., 2021). In addition, most of the studies focused on heterotrophic ciliate species and paid little attention to the mixotrophic part. More specifically, mixotrophs have received scarce attention if any, and are difficult to identify and differentiate with light microscopy or even go unnoticed in fixed material (e.g., small-sized ciliates). The results of this study clearly showed that all these groups are well represented, with several distinct species in different structures of the water column and at different depths. For example, several Strombidiidae ASVs, not reported in other studies focused on microscopy identification, were abundant in this metabarcoding dataset.

A common feature in both metabarcoding and microscopy identification was the temporal variability signal as all groups showed differences between seasons on one hand, and between surface and DCM on the other. During winter, the water column was dominated by heterotrophic forms; species mainly belonged to *Leegaardiella* and *Pelagostrobilidium* genera and most of the Tintinnida were also recorded during this season, whereas during spring and summer, most of the ciliate ASVs detected were annotated as Strombidiida and Tontoniida, which may be mixotrophic (Stoecker et al., 1987; Stoecker et al., 2009; Agatha, 2011). Another interesting differentiation between Choreotrichida on one side, and Strombidiida and Tontoniida on the other, is the vertical distribution. Choreotrichida were mostly recorded at the DCM, while Strombidiida and Tontoniida at the surface. Since, the latter group contains mixotrophic forms, it is possible that they prefer to stay at the surface for the light availability in order to use the chloroplasts sequestered from their prey.

Similarly, signals of seasonality and the shift of the ciliate community composition over the year emerged clearly from several previous studies based on microscopic analyses from different areas of the Mediterranean Sea (Pitta and Giannakourou, 2000; Pitta et al., 2001; Romano and Pitta, 2021; Giner et al., 2019; Romano et al., 2021).

The difference in abiotic variables between HCB and M3A was very low from the seasonal aspect, and did not represent a barrier for marine ciliates. Indeed, most of the ASVs detected in one site were detected also in the other, with the exception of very few ASVs detected only in HCB; for example, two ASVs belonging to *Eutintinnus* sp. (6769 reads), three ASVs belonging to *Strombidium* sp. (1538 total reads), and other rare taxa such as *Eutintinnus apertus* and *Strombidium triquetrum* (987 and 471 total reads, respectively). Those ASVs detected only in HCB belong to groups that are more plastic and adaptable to the high heterogeneity of the coastal stations, which are more affected by different anthropogenic impacts.

Differences in the vertical distribution of plankton communities emerging from the present study confirmed what is known from previous years and studies (Revelante and Gilmartin, 1983; Dolan and Marrasé, 1995; Suzuki and Taniguchi, 1997; Elloumi et al., 2006; Dong et al., 2014; Yang et al., 2014; Yu et al., 2016; Tucker et al., 2017) and may therefore be considered quite typical of the ultra-oligotrophic areas.

While microscopy is still often used in mixoplankton research (Flynn et al., 2019), to investigate the microbial food web dynamics in the marine environment, the metabarcoding approaches allow a more rapid and comprehensive automated examination of environmental samples.

A major novelty of this study is that it provides the first description of the prey-predator co-occurrence network between ciliates and potential prey in two different stations at surface and DCM.

The co-occurrence network in HCB showed a high percentage of depth generalists ASVs at a similar abundance in both surface and DCM, some of which were involved in association with surface and DCM specialists ASVs, like, for example, the positive correlation between the two tintinnids *Amphorellopsis acuta* and *Eutintinnus apertus* with *Ostreococcus* (surface specialist) and *Halosphaera* sp. (DCM specialists), respectively.

Associations between ciliates and Ochrophyta were also observed, and strong positive associations were found between most mixotrophic ciliate species and diatoms. The positive correlations could be explained by cross-feeding or niche overlap (Faust and Raes, 2012); a positive correlation between mixotrophic ciliates and diatoms was also found in studies (Posch et al., 2015) focused on freshwater samples. The positive co-occurrence between mixotrophic ciliates and diatoms suggests a possible niche overlap between these two groups. One hypothesis for this co-appearance was presented by Sonntag et al. (2006), which supported the idea that mixotrophic ciliates are more resistant to solar ultraviolet radiation than heterotrophic ones, thus they can co-exist with big autotrophic cells. In general, mixotrophic ciliate ASVs showed very few negative associations with other potential prey groups in both HCB and M3A. The few exceptions were Strombidiida_F_XX_sp. and Strombidiida_M_X_sp., which were negatively associated with a specific ASV of marine stramenopiles and Streptophytae, respectively, in HCB. On the other hand, heterotrophic ciliates, such as tintinnids, showed negative correlations with different Ochrophyta, Chlorophyta and Cryptophyta. This supports the idea that, in terms of prey selection, mixotrophic ciliates are probably associated to more specific prey in order to retain their chloroplasts, compared to heterotrophic species, such as tintinnids, which are less selective in terms of food. This is also supported by the hypothesis that having many negative interactions is generally interpreted to be the result of interactions like predator-prey relations or allelopathy (Long and Azam, 2001). Positive interactions, on the other hand, indicating groups of organisms having similar, complementary or cooperative functions or activities as well as common preferred environmental conditions (Fuhrman et al., 2008; Eiler et al., 2012), were more evident among the communities of the area.

In our study, it is also important to consider the hubs species that were abundant in M3A. In several studies, hubs have been proposed to be critical or keystone components in a network (Peura et al., 2015; Hernández-Ruiz et al., 2018), but a recent study also demonstrated that known keystone species do not necessarily result in detectable signals in co-occurrence networks (Freilich et al., 2018). The higher number of hubs in M3A and the higher number of connections suggest a higher complexity and species inter-dependence among planktonic protist communities in the open sea. Interactions among species would frequently be enhanced in the open sea as well as in coastal stations and other semi-enclosed areas, possibly due to the proximity to the bottom and to the benthic vegetation or to any other effects deriving from being in confined environments. Different drivers were probably involved in shaping protist communities in HCB, where the number of connections among specialist hubs was considerably lower. Considering also the higher variance explained by environmental variables, a higher influence of abiotic factors seemed to affect the offshore station more. This picture would also be reflected in the annual phytoplankton cycle of HCB, which was more irregular than in M3A, with minor peaks alternating in spring and summer due to the combination of nutrient depletion and sporadic nutrient availability.

## 5 Conclusion

Our study represents a detailed dataset of pelagic ciliate diversity in two stations of the oligotrophic Eastern Mediterranean Sea in four seasons and at two depths.

We investigated the ciliate community based on V4-18S rRNA gene metabarcoding, the first study in the area, increasing knowledge about ciliate diversity not only for groups that have traditionally been neglected (mainly mixotrophs) but also for the main ciliate taxa studied in the long term with morphology-based approaches. The molecular database obtained in this study will serve as a reference for future studies, foster further taxonomic explorations, and will potentially result in an improvement of the quality of studies focused on the dynamics of pelagic ciliates and the relationships they have with the other components of the microbial food web. The co-occurrence approach together with network analysis suggested that heterotrophic and mixotrophic ciliates probably have different ecological niches and are associated with different nano-planktonic groups, suggesting that the relationships with their potential prey differs according to the trophic modes, and this affects the dynamics of the entire microbial food web.

## Data Availability

The data presented in the study are deposited in the NCBI Sequence Read Archive (https://www.ncbi.nlm.nih.gov/sra), accession number: PRJNA990967.

## Footnotes

1
https://support.illumina.com/documents/documentation/chemistry_documentation/16s/16s-metagenomic-library-prep-guide-15044223-b.pdf.
