# Erratum: Community dynamics and co-occurrence relationships of pelagic ciliates and their potential prey at a coastal and an offshore station in the ultra-oligotrophic Eastern Mediterranean Sea

**DOI:** 10.3389/fgene.2023.1358985

**Published:** 2024-01-05

**Authors:** Frontiers Production Office

**Affiliations:** Frontiers Media SA, Lausanne, Switzerland

**Keywords:** barcoding, DNA, mixotrophy, network analysis, prey-predator, vertical/temporal distribution

Due to a production error in **Methods**, **2.2 Filtration**, **DNA extraction and sequencing**, the incorrect access number “PRJNA576330” and the incorrect link https://www.ncbi.nlm.nih.gov/bioproject/PRJNA576330 were used. The correct number is “PRJNA990967”, and the correct link is “https://www.ncbi.nlm.nih.gov/bioproject/PRJNA990967”

The publisher apologizes for this mistake.

The original version of this article has been updated.

