# Supplementary material for: Community dynamics and co-occurrence relationships of pelagic ciliates and their potential prey at a coastal and an offshore station in the ultra-oligotrophic Eastern Mediterranean Sea
Source: Front Genet. 2023 Jul 20;14:1219085. doi: 10.3389/fgene.2023.1219085 (PMC10400710; doi:10.3389/fgene.2023.1219085)
Supplement: Supplementary file 2 [file DataSheet1.docx]

**Figure S1**. Two sampling stations, a coastal (HCB, lat: 35.4342; lon: 25.0792) and an offshore (M3A, lat: 35.7263; lon: 25.1307), which were sampled in March, May, July and October 2019.


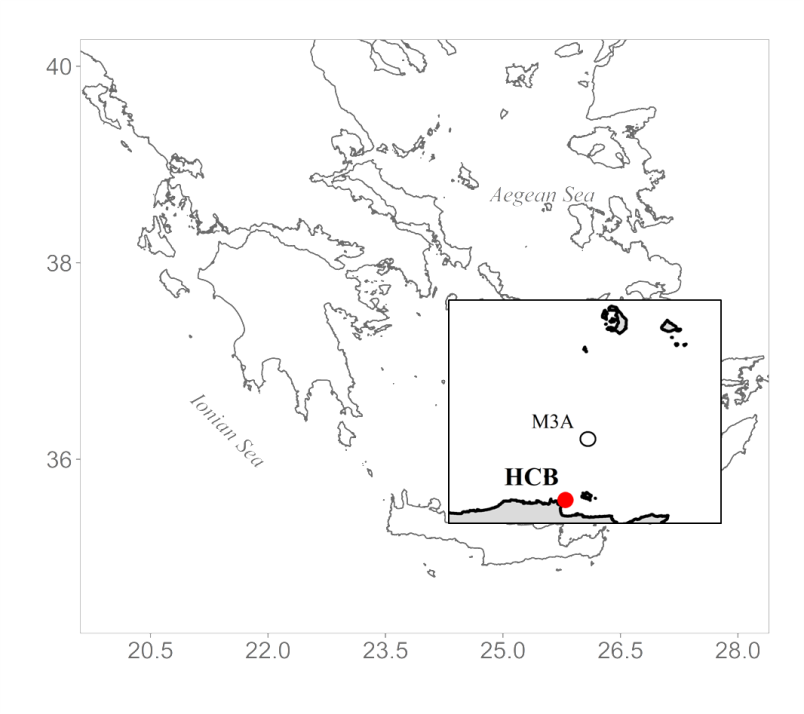


**Figure S2.** Rarefaction curves for all samples under study.

**
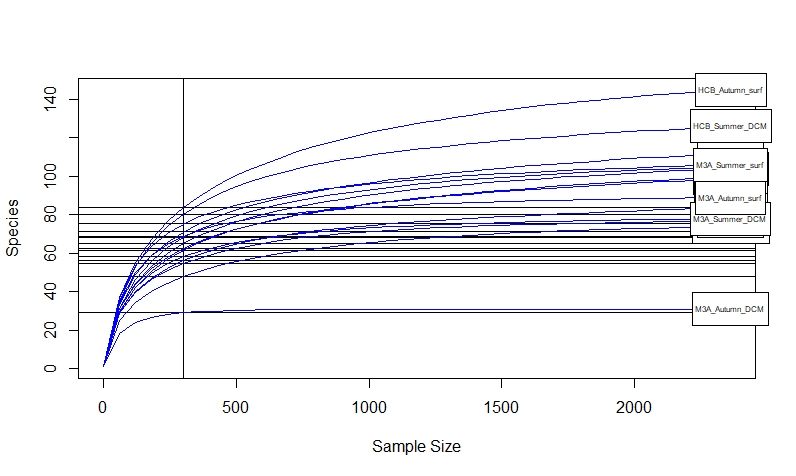
**

**Figure S3**. Alpha diversity on ciliate community structure in different stations, seasons and depths.


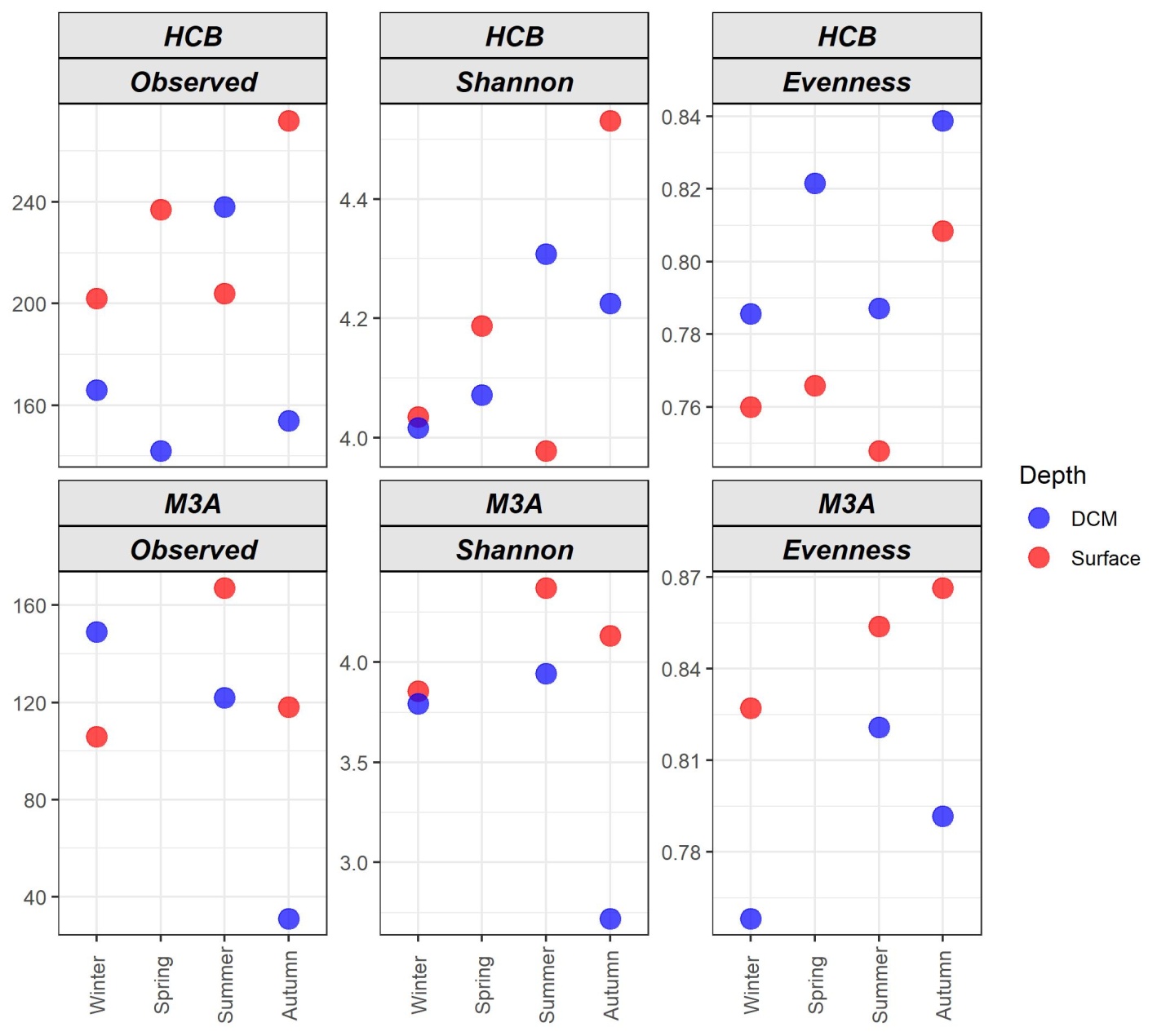


**Figure S4**. NMDS conducted on Bray-Curtis dissimilarity matrix of ciliate community at both stations.


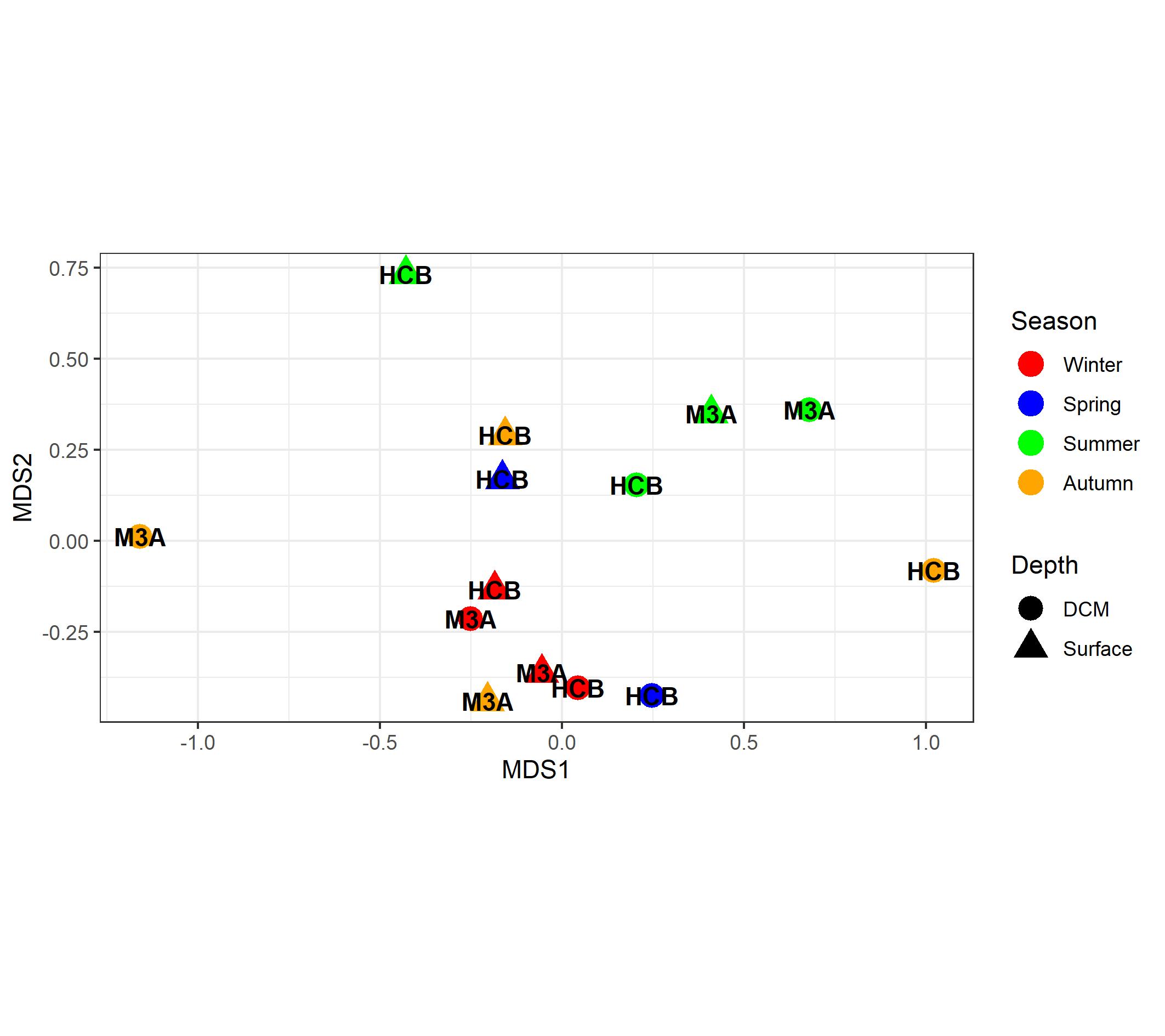


**Figure S5**. Pie chart showing the relative abundance of generalists, specialist surface, specialist DCM and rare species in HCB (coastal) and M3A (offshore). The numbers represent the percentages.
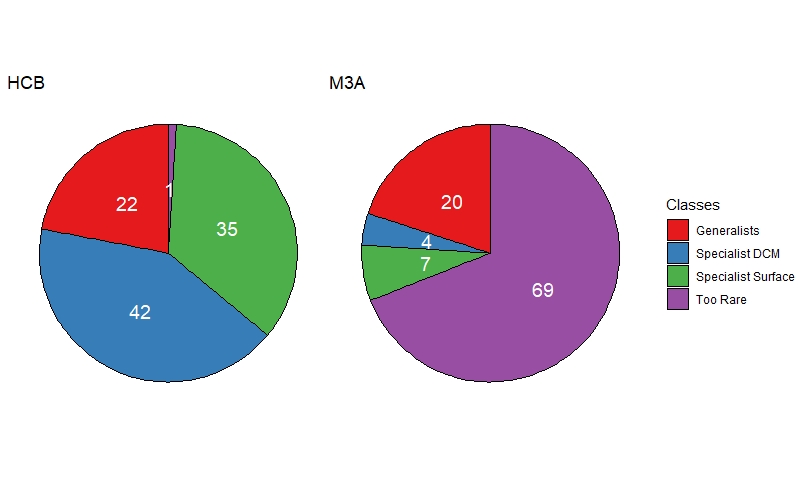


**Table S1**. Temperature, Chlorophyll a (Chla), Dissolved Inorganic Nitrogen (DIN), PO_4_^3-^ (P) and Oxygen (Oxy) values detected in all samples from both HCB (coastal) and M3A (offshore) stations. Highest values are in bold and lowest values are underlined in italic.

| Sample | Station | Season | Layer | Depth | Temperature | Chla | DIN | PO_4_^3-^ |
| --- | --- | --- | --- | --- | --- | --- | --- | --- |
|  |  |  |  | (m) | ^o^C | μg L^-1^ | μΜ | nM |
| HCB_Winter_surf | HCB | Winter | Surface | 2 | *15.4* | **0.51** | **1.92** | **90.33** |
| HCB_Winter_DCM | HCB | Winter | DCM | 50 | 15.9 | 0.40 | 1.38 | 16.00 |
| HCB_Spring_surf | HCB | Spring | Surface | 2 | 19.1 | 0.07 | 0.51 | 8.42 |
| HCB_Spring_DCM | HCB | Spring | DCM | 100 | 17.1 | 0.47 | 1.02 | 7.11 |
| HCB_Summer_surf | HCB | Summer | Surface | 2 | **26.2** | *0.05* | *0.32* | 2.63 |
| HCB_Summer_DCM | HCB | Summer | DCM | 75 | 17.5 | 0.08 | 0.84 | 4.87 |
| HCB_Autumn_surf | HCB | Autumn | Surface | 2 | 22.8 | 0.07 | 0.83 | *2.11* |
| HCB_Autumn_DCM | HCB | Autumn | DCM | 75 | 17.5 | 0.28 | 0.68 | 3.16 |
| M3A_Winter_surf | M3A | Winter | Surface | 2 | *15.3* | **0.43** | 0.84 | 6.00 |
| M3A_Winter_DCM | M3A | Winter | DCM | 50 | 15.6 | 0.37 | 1.10 | 6.42 |
| M3A_Summer_surf | M3A | Summer | Surface | 2 | **25.0** | *0.05* | 0.87 | **26.58** |
| M3A_Summer_DCM | M3A | Summer | DCM | 100 | 16.9 | 0.18 | **1.42** | 26.43 |
| M3A_Autumn_surf | M3A | Autumn | Surface | 2 | 20.2 | 0.10 | *0.55* | *2.10* |
| M3A_Autumn_DCM | M3A | Autumn | DCM | 75 | 17.4 | 0.25 | 0.67 | 3.00 |

**TableS2**. Percentage of reads detected between HCB (coastal station) and M3A (offshore station), between all seasons and between surface and DCM for all ciliate orders. The group “other ciliates” comprises Nassophorea, Prostomatea, and Heterotrichea.

|  | HCB | M3A | Autumn | Winter | Spring | Summer | Surface | DCM |
| --- | --- | --- | --- | --- | --- | --- | --- | --- |
| Other ciliates | 6.19 | 1.95 | 2.64 | 1.13 | 4.04 | 7.65 | 4.65 | 5.82 |
| Strombidiida | 40.84 | 55.47 | 54.37 | 37.79 | 61.20 | 40.55 | 48.59 | 38.32 |
| Tintinnida | 21.08 | 15.99 | 18.26 | 32.00 | 16.13 | 16.75 | 23.73 | 13.65 |
| Choreotrichida | 31.89 | 26.59 | 24.73 | 29.08 | 18.63 | 35.05 | 23.03 | 42.21 |

**TableS3**. Results from MANOVA analysis between stations, seasons and depths.

| Response Observed : |  |  |  |  |  |  |
| --- | --- | --- | --- | --- | --- | --- |
|  | Df | Sum Sq | Mean Sq | F value | *p* |  |
| Station | 1 | 25579.30 | 25579.30 | 13.22 | 0.01 | ** |
| Season | 3 | 3600.40 | 1200.10 | 0.62 | 0.62 |  |
| Depth | 1 | 6601.10 | 6601.10 | 3.41 | 0.10 |  |
| Residuals | 8 | 15476.90 | 1934.60 |  |  |  |
| --- |  |  |  |  |  |  |
| Response Shannon : |  |  |  |  |  |  |
|  | Df | Sum Sq | Mean Sq | F value | *p* |  |
| Station | 1 | 0.46 | 0.46 | 2.58 | 0.15 |  |
| Season | 3 | 0.15 | 0.05 | 0.29 | 0.83 |  |
| Depth | 1 | 0.29 | 0.29 | 1.63 | 0.24 |  |
| Residuals | 8 | 1.43 | 0.18 |  |  |  |
|  |  |  |  |  |  |  |
| Response Evenness : |  |  |  |  |  |  |
|  |  |  |  |  |  |  |
|  | Df | Sum Sq | Mean Sq | F value | *p* |  |
| Station | 1 | 0.00 | 0.00 | 1.34 | 0.28 |  |
| Season | 3 | 0.00 | 0.00 | 0.18 | 0.91 |  |
| Depth | 1 | 0.00 | 0.00 | 1.12 | 0.32 |  |
| Residuals | 8 | 0.00 | 0.00 |  |  |  |
|  |  |  |  |  |  |  |
|  | | | |  |  |  |

**Table S4**. Values of Observed ASVs, Shannon and Pielou’s Evenness indices in all samples. The highest values are in bold, while the lowest values are underlined in italics.

| **Sample** | **Station** | **Season** | **Depth** | **Observed** | **Shannon** | **Evenness** |
| --- | --- | --- | --- | --- | --- | --- |
| HCB_Winter_surf | HCB | Winter | Surface | 202 | 4.03 | 0.76 |
| HCB_Winter_DCM | HCB | Winter | DCM | 166 | 4.02 | 0.79 |
| HCB_Spring_surf | HCB | Spring | Surface | 237 | 4.19 | 0.77 |
| HCB_Spring_DCM | HCB | Spring | DCM | *142* | 4.07 | 0.82 |
| HCB_Summer_surf | HCB | Summer | Surface | 204 | *3.98* | *0.75* |
| HCB_Summer_DCM | HCB | Summer | DCM | 238 | 4.31 | 0.79 |
| HCB_Autumn_surf | HCB | Autumn | Surface | **272** | **4.53** | 0.81 |
| HCB_Autumn_DCM | HCB | Autumn | DCM | 154 | 4.23 | **0.84** |
| M3A_Winter_surf | M3A | Winter | Surface | 106 | 3.86 | 0.83 |
| M3A_Winter_DCM | M3A | Winter | DCM | 149 | 3.79 | *0.76* |
| M3A_Summer_surf | M3A | Summer | Surface | **167** | **4.37** | 0.85 |
| M3A_Summer_DCM | M3A | Summer | DCM | 122 | 3.94 | 0.82 |
| M3A_Autumn_surf | M3A | Autumn | Surface | 118 | 4.13 | **0.87** |
| M3A_Autumn_DCM | M3A | Autumn | DCM | *31* | *2.72* | 0.79 |
